# Roles of Lymphocyte Kv1.3-Channels in the Pathogenesis of Renal Diseases and Novel Therapeutic Implications of Targeting the Channels

**DOI:** 10.1155/2015/436572

**Published:** 2015-03-18

**Authors:** Itsuro Kazama

**Affiliations:** Department of Physiology I, Tohoku University Graduate School of Medicine, Seiryo-cho, Aoba-ku, Sendai, Miyagi 980-8575, Japan

## Abstract

Delayed rectifier K^+^-channels (Kv1.3) are predominantly expressed in T lymphocytes. Based on patch-clamp studies, the channels play crucial roles in facilitating the calcium influx necessary to trigger lymphocyte activation and proliferation. Using selective channel inhibitors in experimental animal models, *in vivo* studies then revealed the clinically relevant relationship between the channel expression and the pathogenesis of autoimmune diseases. In renal diseases, in which “chronic inflammation” or “the overstimulation of cellular immunity” is responsible for the pathogenesis, the overexpression of Kv1.3-channels in lymphocytes promotes their cellular proliferation and thus contributes to the progression of tubulointerstitial fibrosis. We recently demonstrated that benidipine, a potent dihydropyridine calcium channel blocker, which also strongly and persistently inhibits the lymphocyte Kv1.3-channel currents, suppressed the proliferation of kidney lymphocytes and actually ameliorated the progression of renal fibrosis. Based on the recent *in vitro* evidence that revealed the pharmacological properties of the channels, the most recent studies have revealed novel therapeutic implications of targeting the lymphocyte Kv1.3-channels for the treatment of renal diseases.

## 1. Introduction

**Figure 1:**
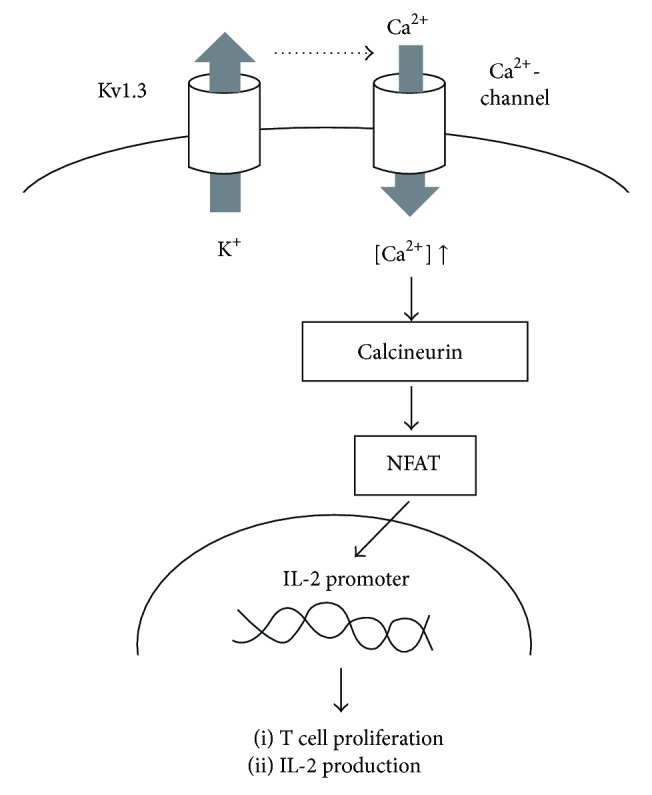
Kv1.3-channel-induced activation pathway of T lymphocytes. Kv1.3-channels expressed in T lymphocytes facilitate the calcium influx necessary to trigger the lymphocyte activation and proliferation. The rise in the intracellular calcium concentration stimulates the phosphatase calcineurin activity, which then dephosphorylates nuclear factor of activated T cells (NFAT), enabling it to accumulate in the nucleus and bind to the promoter of the gene encoding interleukin 2 (IL-2).

T lymphocytes predominantly express delayed rectifier K^+^-channels (Kv1.3) in their plasma membranes [1–3]. Using selective channel inhibitors, patch-clamp studies revealed that the channels generate the K^+^-diffusion potential across the plasma membranes and play crucial roles in facilitating calcium influx necessary to trigger the lymphocyte activation and proliferation [3–6]. Previous studies demonstrated the involvement of inflammatory leukocytes, such as T lymphocytes, macrophages, and mast cells, in the pathogenesis of renal diseases, such as glomerulonephritis, chronic kidney disease (CKD), or tubulointerstitial fibrosis [7–11]. Since lymphocytes are actually activated [12] and serum cytokine levels are known to be elevated in patients with advanced-stage renal diseases [13, 14], Kv1.3-channels expressed in lymphocytes would contribute to the progression of the diseases. Regarding the molecular mechanisms by which lymphocytes are activated, the rise in the intracellular calcium concentration stimulates the phosphatase calcineurin activity, which then dephosphorylates nuclear factor of activated T cells (NFAT), enabling it to accumulate in the nucleus and bind to the promoter of the gene encoding interleukin 2 (IL-2) [6, 15] (Figure 1). Therefore, pharmacological targeting of calcineurin has been the main mechanism by which drugs, such as cyclosporine and tacrolimus, exert their immunosuppressive effects [16]. However, recent studies have also revealed that selective inhibition of lymphocyte Kv1.3-channels also represses lymphocyte activity and thus suppresses cellular immunity [17]. Recent patch-clamp studies, including ours, have shown that commonly used drugs, such as calcium channel blockers (CCBs) [18, 19], macrolide antibiotics, and HMG-CoA reductase inhibitors, effectively suppress the Kv1.3-channel currents in lymphocytes [20, 21]. Such studies suggested the therapeutic efficacy of these drugs for the treatment of renal diseases, in which “chronic inflammation” or “the overstimulation of cellular immunity” is responsible for the pathogenesis [22]. By summarizing the previous and recent findings obtained from studies in the relevant fields, this review provides an overview of the pathological roles of lymphocyte Kv1.3-channels in renal diseases. Based on the recent* in vitro *and* in vivo* evidence that revealed the pharmacological properties of the channels, this review also focuses on the novel therapeutic implications of targeting the channels for the treatment of renal diseases.

## 2. Increased Numbers of Leukocytes in Rat Kidneys with Renal Diseases

**Figure 2:**
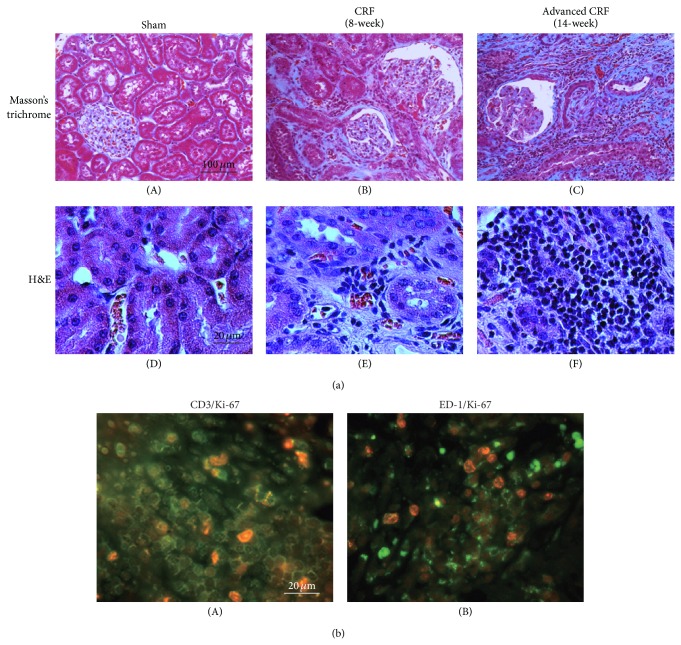
Histological features of sham-operated (sham), CRF, and advanced CRF rat kidneys. (a) Hematoxylin and eosin (H&E) and Masson's trichrome staining, in sham-operated (sham), CRF (8 weeks after nephrectomy), and advanced CRF (14 weeks after nephrectomy) rat kidneys. ((A), (B), and (C)) Low-power views of cortex, Masson's trichrome. Magnification, ×20. ((D), (E), and (F)) High-power views of cortical interstitium, H&E. Magnification, ×60. (b) Immunofluorescence staining using antibodies for CD3 ((A) green) and ED-1 ((B) green), costained with Ki-67 (red) in advanced CRF rat kidneys. High-power views of the cortical interstitium. Magnification, ×60. Modified from [34].

Previous studies have described several laboratory models of renal diseases, including ligation of the renal artery branches or unilateral ureter [23, 24], ablation of renal mass by surgery [25, 26], toxic nephritis [27, 28], and immunologically induced nephritis [29, 30]. In the development of glomerulonephritis, inflammatory leukocytes are initially recruited from the bone marrow and infiltrate into the renal interstitium to produce proinflammatory cytokines [9]. Therefore, the kidneys from rat models with toxic or immunologically induced nephritis were characterized by the massive infiltration of T-lymphocytes or macrophages [9, 27–30]. On the other hand, in rat models with 5/6 nephrectomy (subtotal nephrectomy), the injured kidneys were mainly characterized by severe glomerulosclerosis, which was primarily caused by the renal hemodynamic changes, such as the increased glomerular pressure and the protein overload [31, 32]. However, with the increase in the serum creatinine, the kidneys from these subtotally nephrectomized rats were additionally characterized by diffuse interstitial fibrosis with the involvement of leukocyte infiltration [7, 8, 33]. In rats with subtotal nephrectomy followed by longer recovery periods, serum creatinine and blood urea nitrogen levels were markedly elevated, indicating advanced chronic renal failure (CRF) [11, 34]. In CRF rat kidneys with 8-week recovery period, the cortical interstitium was expanded with fibroedema (Figure 2(a)(B) versus Figure 2(a)(A)) and there was some infiltration of small round cells among spindle-shaped fibroblasts (Figure 2(a)(E) versus Figure 2(a)(D)). At 14 weeks, in addition to diffuse fibrosis in the cortical and medullary interstitium (Figure 2(a)(C)), the numbers of small round cells were dramatically increased in the cortical interstitium (Figure 2(a)(F)). Since the cortical expression of CD3 and ED-1, surface markers for T lymphocytes and macrophages, was markedly elevated [11], they were regarded as inflammatory leukocytes, such as T lymphocytes and macrophages. By immunohistochemistry, the CD3- or ED-1-positive small round cells were actually costained with Ki-67, a marker of cellular proliferation (Figures 2(b)(A) and 2(b)(B)). The findings indicated that T lymphocytes and macrophages were proliferating prominently within the cortical interstitium of advanced CRF rat kidneys [34].

Recently, Liu et al. demonstrated that CD4^+^ T-lymphocytes, especially Th2 cells, contributed to the progression of renal fibrosis in a rat model of unilateral ureteral obstruction (UUO) [35]. In the development of the tubulointerstitial fibrosis in CRF rat kidneys, previous studies demonstrated that the inflammatory leukocytes were initially recruited from the bone marrow and infiltrated into the renal interstitium to trigger the proliferation of fibroblasts [36]. In this context, using the rat model of advanced CRF, the recent studies further demonstrated that the infiltrated leukocytes proliferated* in situ *in the cortical interstitium, and thus dramatically increased their numbers [11, 34].

## 3. Physiological Roles of Kv1.3-Channels Expressed in T-Lymphocytes

**Figure 3:**
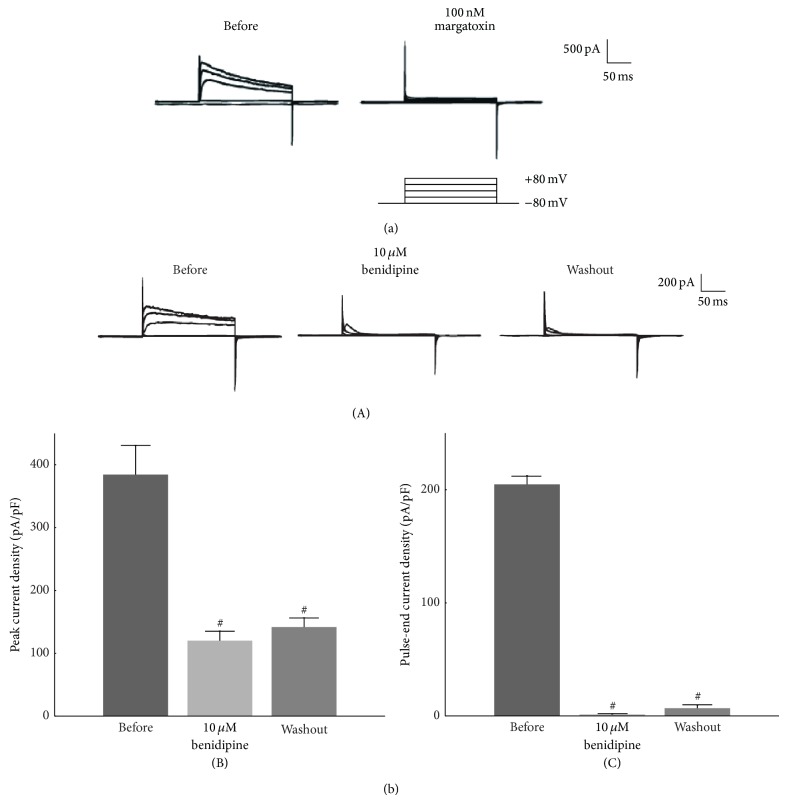
Effects of margatoxin and benidipine on Kv1.3 channel currents in murine thymocytes. Typical whole-cell current traces at different voltage steps recorded before and after the application of 100 nM margatoxin (a) or 10 *μ*M benidipine ((b)(A)). The currents were elicited by voltage-steps from the holding potential of −80 mV to −40, 0, 40, and 80 mV, as depicted in the voltage protocol. Each pulse was applied for a 200-ms duration between 10-second intervals. (b)(B) Peak current densities (peak currents normalized by the membrane capacitance) obtained from the records in (A) at the voltage-step of 80 mV. (b)(C) Pulse-end current densities (pulse-end currents normalized by the membrane capacitance) obtained from the records in (a) at the voltage-step of 80 mV. ^#^ *P* < 0.05 versus before the drug application. Values are means ± SEM (*n* = 5). Differences were analyzed by ANOVA followed by Dunnett's or Student's *t*-test. Modified from [18, 38].

A variety of ion channels that are expressed in T lymphocytes include voltage-dependent K^+^-channels (Kv), Ca^2+^-activated K^+^-channels (K_Ca_), Ca^2+^ release-activated Ca^2+^-channels (CRAC), Mg^2+^-inhibited Ca^2+^-permeable current (MIC) channels, and swelling-activated Cl^−^ channels (Cl_swell_) [37]. Among them, human T lymphocytes predominantly express Kv1.3-channels in their plasma membranes [1, 2]. In patch-clamp studies using thymus-derived murine lymphocytes (thymocytes), stepwise changes in the membrane potential evoked membrane currents characteristic to Kv-channels [18, 20, 21, 38, 39]. Since margatoxin, a selective inhibitor of Kv1.3-channels, almost totally abolished the currents in lymphocytes (Figure 3(a)) [38], these membrane currents were identified as Kv1.3-channel currents. The physiological roles of Kv1.3-channels in lymphocytes have been identified by several* in vitro *studies. Patch-clamp studies revealed that these channels generate the K^+^-diffusion potential across the plasma membranes and thus play a role in regulating the resting membrane potential and controlling the cell volume [40, 41]. The opening of Kv1.3-channels also brings about the membrane hyperpolarization and generates a driving force for the Ca^2+^ influx [4, 5, 37]. Consequently, it stimulates the following Ca^2+^ signaling pathway necessary to trigger the lymphocyte activation and proliferation.

## 4. Pathological Roles of Lymphocyte Kv1.3-Channels in Renal Diseases

Peripheral lymphocytes are activated and the serum cytokine levels are known to be elevated in patients with end-stage renal disease [12–14]. Based on a previous patch-clamp study [42], the conductance of voltage-dependent K^+^-channels in lymphocytes was increased in such patients and the activity of the channels was strongly associated with the severity of renal dysfunction. In experimental animal models of renal diseases, such as renal allograft rejection [10] and glomerulonephritis [9], immunosuppression by the blockade of lymphocyte Kv1.3-channels actually prevented or ameliorated the progression of the diseases. By using selective Kv1.3-channel inhibitors, such as ShK and Psora-4, therapeutically [9, 10], these studies demonstrated the contribution of the channels to the pathogenesis of renal diseases.

In previous studies, the overexpression of Kv1.3-channnels was demonstrated in cells under various pathologic conditions, such as cancer [43, 44], ischemic heart disease [45], and autoimmune disorders [46, 47]. In autoimmune disorders, including multiple sclerosis, rheumatoid arthritis, and systemic lupus erythematosus, the inhibition of the Kv1.3-channel modulated the calcium influx patterns in T lymphocytes and thus exerted immunosuppressive effects [48–50]. Recently, using a rat model with advanced CRF, we demonstrated for the first time that Kv1.3-channels were overexpressed in proliferating leukocytes within fibrotic kidneys [11]. In our study, margatoxin, one of the most potent inhibitors of Kv1.3-channels which almost totally inhibits the channel currents in lymphocytes (Figure 3(a)) [38], decreased the number of proliferating lymphocytes and ameliorated the progression of renal fibrosis. These findings indicated that the overexpression of Kv1.3-channels in kidney lymphocytes promoted their cellular proliferation in advanced CKD. As previously demonstrated in cancer cells [51], the membrane hyperpolarization induced by the overexpression of the channels is thought to trigger the cell cycle progression in lymphocytes [52, 53]. Since the cytokines produced by lymphocytes stimulate the activity of fibroblasts to produce collagen [36], the proliferating lymphocytes in the interstitium would promote the progression of renal fibrosis in advanced CRF, contributing to the rapid deterioration of renal function [11].

## 5. Suppressive Effects of Benidipine on Lymphocyte Kv1.3-Channels

In addition to their cardiovascular effects on hypertension and ischemic heart disease [54], CCBs are also known to exert immunosuppressive properties in humans [55, 56]. According to several* in vitro* studies, CCBs, including nifedipine, verapamil, and diltiazem, repress the migration of leukocytes and inhibit their proliferation [57, 58]. Recently, using human peripheral leukocytes, Matsumori et al. demonstrated that CCBs also suppress the production of proinflammatory cytokines, such as IL-1*β*, tumor necrosis factor *α* (TNF-*α*), and interferon *γ* (IFN-*γ*) [59, 60]. According to their studies, 1,4-dihydropyridine (DHP) CCBs, including nifedipine, amlodipine, and benidipine, which are highly lipophilic compared to the other types of CCBs [61, 62], exert relatively stronger immunomodulatory effects. Among them, benidipine is one of the most lipophilic and longest acting DHP CCBs [63, 64]. In our patch-clamp study using murine thymocytes [18], benidipine almost totally and irreversibly inhibited the Kv1.3-channel currents (Figure 3(b)), indicating its usefulness as a potent Kv1.3-channel inhibitor. Although the effects of benidipine on cytokine production have not yet been directly examined [59, 60], the marked inhibition of the channel currents by this drug strongly suggested its higher immunosuppressive potency than the other CCBs. Moreover, the persistent effect of benidipine in decreasing the channel currents may suggest its longer duration of action in thymocytes, as previously demonstrated in cardiomyocytes [64] and isolated coronary arteries [65].

## 6. Therapeutic Efficacy of Benidipine against the Progression of Tubulointerstitial Fibrosis in Advanced CRF

In our recent study, the overexpression of Kv1.3-channels in kidney lymphocytes was strongly associated with their* in situ* proliferation in advanced CRF rat kidneys [11], and the channel inhibition by margatoxin actually decreased the number of infiltrating leukocytes and slowed the progression of renal fibrosis. Based on one of our patch-clamp studies [18], since benidipine was also highly potent as a Kv1.3-channel inhibitor, the therapeutic effects of this drug on CKD were examined in our most recent study [34]. Previous studies have shown the therapeutic efficacy of antihypertensive drugs, such as CCBs [66] and angiotensin converting enzyme inhibitors (ACEIs) [67], for the prevention of glomerulosclerosis, since these drugs hemodynamically ameliorate glomerular hypertension. However, it is not well known how these pharmacological approaches slow the progression of tubulointerstitial fibrosis independently of their effects on glomerulosclerosis.

**Figure 4:**
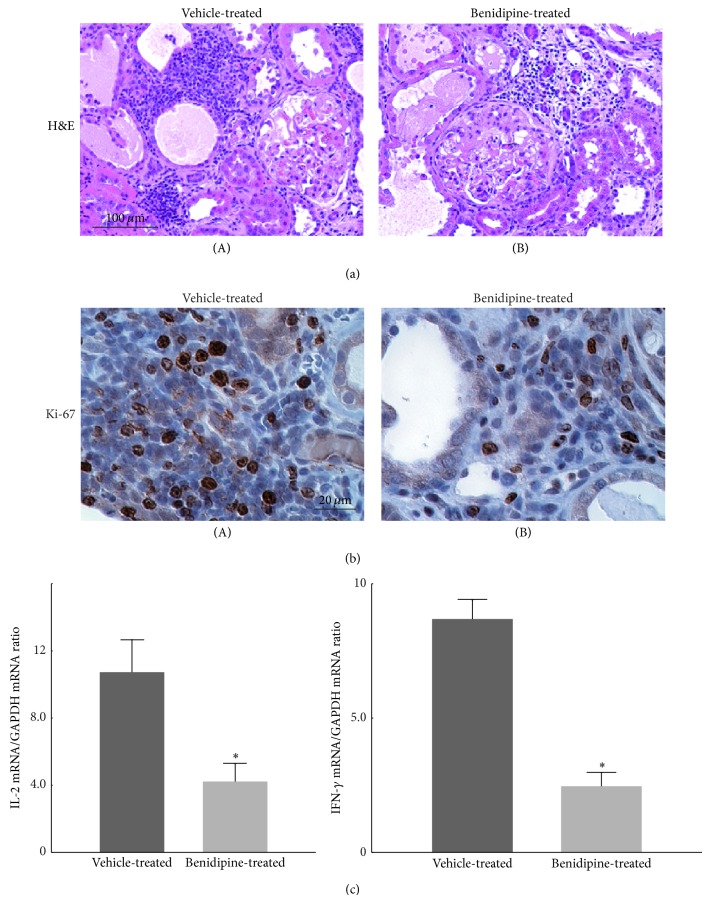
Histological features of vehicle- and benidipine-treated CRF rat kidneys and the expression of proinflammatory cytokines. (a) Hematoxylin and eosin staining (H&E) in vehicle-treated and benidipine-treated CRF rat kidneys. Low-power views of the cortex. Magnification, ×20. (b) Immunohistochemistry using antibody for Ki-67 (brown) in vehicle- and benidipine-treated CRF rat kidneys. High-power views of the cortical interstitium. Magnification, ×60. (c) The abundance of IL-2 mRNA (left) and interferon-*γ* (IFN-*γ*) (right) in the renal cortex of vehicle-treated and benidipine-treated CRF rat kidneys. ^*^ *P* < 0.05 versus vehicle-treated CRF rats. Values are means ± SEM (*n* = 5). Differences were analyzed by ANOVA followed by Dunnett's or Student's *t*-test. Modified from [34].

**Figure 5:**
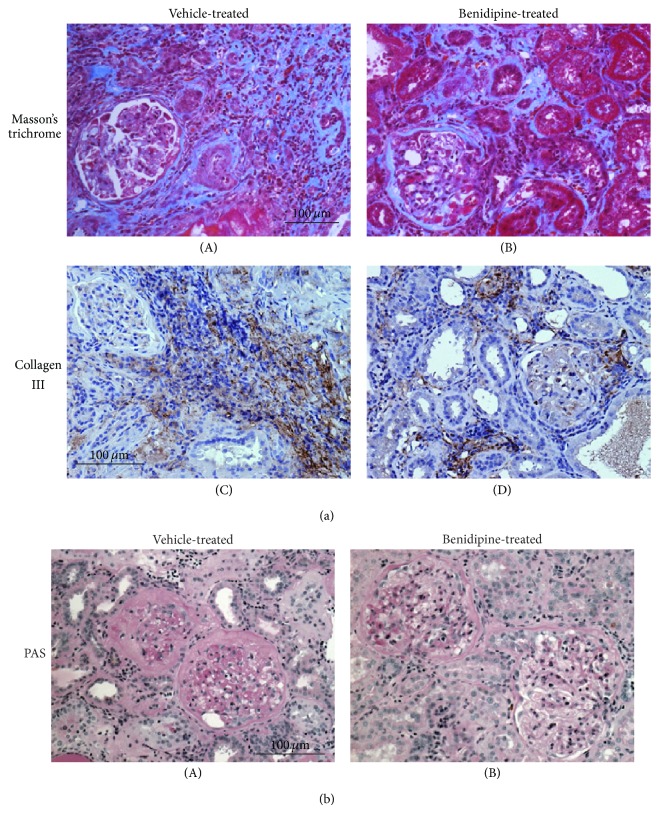
Fibrotic marker expression and histological features of glomeruli in vehicle- and benidipine-treated CRF rat kidneys. (a) Masson's trichrome staining ((A) and (B)) and immunohistochemistry using antibody for collagen III ((C) and (D) brown) in vehicle-treated and benidipine-treated CRF rat kidneys. Low-power views of the cortex. Magnification, ×20. (b) Periodic acid Schiff (PAS) staining in vehicle-treated and benidipine-treated CRF rat kidneys. Low-power views of the cortex. Magnification, ×20. Modified from [34].

In advanced CRF rat kidneys with benidipine treatment, the size of the cortical interstitium, which included the areas of fibrosis, edema, and the inflammatory leukocyte infiltration, was smaller (Figure 4(a)(B) versus Figure 4(a)(A)), and the number of proliferating leukocytes was much reduced (Figure 4(b)(B) versus Figure 4(b)(A)) together with a significant decrease in the proinflammatory cytokine expression (Figure 4(c)). In these kidneys, Masson's trichrome staining and the immunohistochemistry for fibrosis markers, such as collagen III, demonstrated less staining in the cortical interstitium (Figures 5(a)(B) and 5(a)(D) versus Figures 5(a)(A) and 5(a)(C)). However, in the glomeruli, the amount of periodic acid Schiff positive material was a comparable with that in vehicle-treated CRF rat kidneys (Figure 5(b)(B) versus Figure 5(b)(A)). These results indicated that benidipine ameliorated the progression of renal fibrosis without affecting the deterioration of glomerulosclerosis. Benidipine, which blocks L- and T-types of calcium channels in the renal vasculature, is known to dilate both afferent and efferent arterioles of the glomeruli and to reduce glomerular hypertension [68]. In our recent study, however, benidipine did not decrease the severe proteinuria nor did it improve the systemic hypertension in advanced CRF rats [34]. Therefore, factors other than reducing the glomerular capillary pressure may also be involved in its pharmacological effects of ameliorating the renal injury [66]. Recently, benidipine was shown to reduce the circulating levels of inflammatory cytokines or proteins, such as IL-6 and high mobility group box-1 (HMGB-1), in patients with chronic kidney disease [69]. Since IL-6 and HMGB-1 accelerate the inflammation of systemic organs by promoting lymphocyte activation and proliferation [14, 70]; such effects of benidipine may have therapeutic potential for slowing the progression of renal fibrosis in advanced CRF.

## 7. Novel Therapeutic Implications of Targeting Lymphocyte Kv1.3-Channels by Commonly Used Drugs

**Figure 6:**
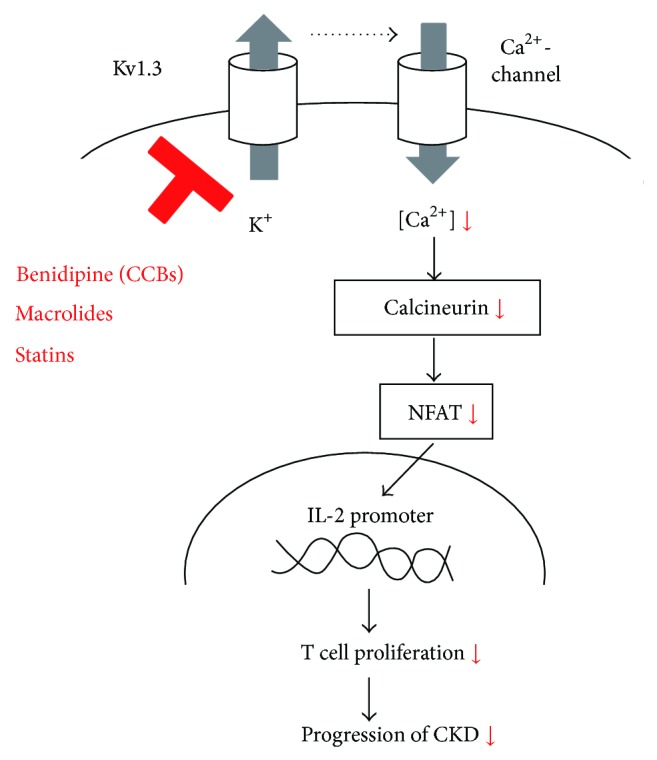
Therapeutic implications of targeting lymphocyte Kv1.3-channels in the treatment of CKD. In addition to benidipine, one of the calcium channel blockers (CCBs), macrolide antibiotics, and statins, which effectively inhibit lymphocyte Kv1.3-channels, could also potentially be useful in the treatment or the prevention of chronic kidney disease (CKD).

Grgic et al. demonstrated the therapeutic efficacy of blocking the intermediate-conductance Ca^2+^-activated K^+^-channels (K_Ca_3.1) for renal fibrosis, since fibroblasts overexpressed the channels under a pathologic condition [24]. In a separate animal study, they also demonstrated the prophylactic efficacy of blocking lymphocyte Kv1.3-channels to prevent renal allograft rejection [10]. As an extension of these studies, our studies further suggested that targeting the Kv1.3-channels overexpressed in leukocytes would also be useful for the treatment of renal fibrosis in advanced CRF [11, 34]. In our series of patch-clamp studies, in addition to CCBs, such as benidipine and nifedipine [18], macrolide antibiotics and HMG-CoA reductase inhibitors (statins) also effectively suppressed lymphocyte Kv1.3-channel currents [20, 21] (Table 1). According to separate* in vitro *studies, these drugs exerted immunomodulatory properties besides their anti-inflammatory, antimicrobial, and anticholesterol effects [56, 71–75]. Since lymphocyte Kv1.3-channels trigger calcium influx, which is necessary for IL-2 synthesis [15] and since channel blockade by highly selective inhibitors, including margatoxin, ShK-Dap^22^, and PAP-1, actually repressed the immune response in lymphocytes [38, 76–78] (Table 2), the suppressive effects of NSAIDs, macrolide antibiotics, and statins on the channel currents were considered to contribute to their immunomodulatory properties. Additionally, as previously detected by the whole-cell patch-clamp technique [38], the amplitude of the peak channel currents was deeply associated with the “activation” of the channel currents [21]. Therefore, the stronger suppression of the peak currents by macrolide antibiotics or statins may indicate their higher immunosuppressive potency. In this regard, besides the use of the previously developed selective blockers for the channels [78–80], the use of CCBs, macrolide antibiotics, or statins could also potentially be useful as antifibrotic agents in patients with advanced CKD (Figure 6). The most likely side effects of the Kv1.3-channel inhibition include epileptic seizures or enteric twitches, since the channels are also expressed in the synapses of both central and enteric nervous systems [81, 82]. In normal rat kidneys, Kv1.3-channels are expressed in some proximal tubules [9] and predominantly in the basolateral membranes of the inner medullary collecting duct cells [83]. In these cells, the channels regulated the cellular or total body fluid volume by maintaining the driving force for Na^+^ reabsorption. Therefore, the channel inhibition may also affect such tubular functions and the urinary K^+^ secretion, which was actually demonstrated in previous studies using isolated collecting ducts [84]. However, compared to the highly selective inhibitors, which were originally derived from venom, scorpion, or sea anemone peptide toxins [49, 79, 85, 86], the drugs, such as CCBs, macrolide antibiotics, or statins, could be used more safely, since they have been employed in a common clinical practice for a longer period of time.

## 8. Conclusions and Perspectives

In a physiological condition, Kv1.3-channels expressed in T lymphocytes play crucial roles in the initiation of the immune response. In renal diseases, such as CKD, acute glomerulonephritis, and renal allograft rejection, the channels contribute to the pathogenesis of the diseases. In rat kidneys with advanced CRF, the overexpression of the channels in lymphocytes facilitated the progression of tubulointerstitial fibrosis by promoting lymphocyte proliferation, suggesting that the channel could be a potent therapeutic target for advanced-stage CKD. Benidipine, one of the most commonly used CCBs, which also strongly and persistently inhibits the lymphocyte Kv1.3-channel currents, suppressed the proliferation of kidney lymphocytes and actually ameliorated the progression of renal fibrosis. Since other drugs, such as NSAIDs, macrolide antibiotics, and statins, also effectively suppress the channel currents in lymphocytes, they may be useful for treating or preventing renal diseases.

“Chronic inflammatory diseases” are a category of diseases, in which “chronic inflammation” or “the overstimulation of cellular immunity” is responsible for the pathogenesis [22]. Besides infectious diseases and autoimmune disorders, a number of diseases, such as cancer, neuroinflammatory diseases, and metabolic disorders, nowadays fall into this category [87]. Recently, in addition to renal diseases, the involvement of lymphocyte Kv1.3-channels has also been demonstrated in the development of these “chronic inflammatory diseases” [43–45, 48–50, 52, 88–91]. Therefore, our future tasks would include revealing the as yet unknown significance of the channels in the pathogenesis of these diseases and revealing novel therapeutic applications based on targeting these channels.

## Figures and Tables

**Table 1:** Summary of changes in peak and pulse-end currents after application of CCBs, macrolide, and HMG-CoA reductase inhibitors (statins).

Drugs	*N*	Peak current density (pA/pF)		Pulse-end current/peak current (*I*/*I*_peak_) (%)	
		Before	After	Before	After
Nifedipine (100 *μ*M)	5	297 ± 3.3	196 ± 2.6^#^	61.3 ± 1.8	26.2 ± 5.4^#^
Benidipine (10 *μ*M)	5	384 ± 47	120 ± 15^#^	53.4 ± 4.6	1.14 ± 0.21^#^
Clarithromycin (100 *μ*M)	5	277 ± 4.4	89.6 ± 10^#^	48.5 ± 1.4	15.8 ± 1.0^#^
Pravastatin (1 mM)	5	309 ± 16	278 ± 17	52.1 ± 2.4	33.4 ± 4.2^#^
Lovastatin (10 *μ*M)	5	301 ± 12	237 ± 5.0^#^	55.1 ± 0.4	26.8 ± 0.7^#^
Simvastatin (10 *μ*M)	5	307 ± 3.7	225 ± 28^#^	47.3 ± 3.9	12.1 ± 2.2^#^

Data modified from [18, 20, 21].

Values are means ± SEM.

CCBs: calcium channel blockers.

^#^ *P* < 0.05 versus before drug application.

**Table 2:** Summary of Kv1.3-channel inhibition and its functional outcomes.

Authors	Cells	Kv1.3-channel inhibitors used	Functional outcomes of Kv1.3-channel inhibition
Villalonga et al. [76]	Raw 264.7 macrophages Jurkit T-lymphocytes	Margatoxin Charybdotoxin	Immunomodulatory effect
Kalman et al. [77]	L929 cell line stably expressing Kv1.3	ShK-Dap^22^	Immunomodulatory effect
Schmitz et al. [78]	L929 cell line stably expressing Kv1.3	PAP-1	Immunomodulatory effect
Kazama et al. [38]	Murine thymocytes	Margatoxin	Membrane stabilization Immunomodulatory effect
Leanza et al. [80]	Cancer cell lines (MCF-7, DLD-1 etc.)	PAP-1 Psora-4	Cellular proliferation Apoptosis regulation
Hamilton et al. [79]	Skeletal muscle cell lines (C_2_C_12_, L6)	Margatoxin PAP-1 Psora-4	Increased glucose uptake Increased AMPK activity

ShK: *Stichodactyla helianthus *toxin; PAP-1: 5-(4-phenylbutoxy) psoralen.

AMPK: 5′ adenosine monophosphate-activated protein kinase.
